# Abuse of Cocaine

**Published:** 1893-03

**Authors:** 


### ABUSE OF COCAINE.

Almost everything that is of use to man is capable of abuse. This is especially true of stimulants and sedatives. These drugs in their elementary state, are generally violent poisons. Even tea and coffee are not exceptions to the rule. The abuse of such things consists in using them too much, or for improper purposes. Nature meant them for medicines, and used intelligently and carefully as such, they are among her best gifts to the afflicted.

Cocaine, obtained from the elementary principle of coca leaves, is exceedingly valuable in minor surgical operations as a substitute for ether and chloroform; but already it is becoming fearfully abused. According to the London *Lancet*, approving a paper on the subject in the *Journal of Mental Science*, its special dangers are three: it is treacherous; it produces an early breakdown, both morally and intellectually; it is intensely poisonous, and speedily causes destructive tissue changes.

In chronic cocaine poisoning, general wasting appears early and develops with extreme rapidity. Convulsions also are not uncommon. In animals it is found to produce degeneration in the cells of the medulla and spinal cord, and also in the nerve cells of the heart ganglia and in the liver cells.

The great danger of cocaine lies in the fact that it is the most agreeable and alluring of all narcotics. It causes no mental confusion, only a little more talkativeness than usual. There is no headache or nausea, and the pleasant effects are produced with a comparatively small dose ; but symptoms of poisoning are rapidly developed, and within three months of the commencement of the habit there may be marked indications of degeneration, loss of memory, hallucinations and suspicions.

The author of the paper in the *Journal of Mental Science*, says that much harm has resulted from a recent tendency to use cocaine to break off the opium habit, and from a mistaken notion that this drug can be employed safely and advantageously for that purpose. The writer adds that cocaine is more insidious than morphine, fastens more readily upon its victim and holds him in at least as tight a grasp.— *Youths Companion.*

